# Supplementary material for: Splice-Junction-Based Mapping of Alternative Isoforms in the Human Proteome
Source: Cell Rep. Author manuscript; Available in PMC 2020 Jan 15. (PMC6961840; doi:10.1016/j.celrep.2019.11.026)
Supplement: 2 [file NIHMS1546469-supplement-2.zip › DF1/PXD006675/PulmonaryVein/PulmonaryVein_5_TTN_APHVEFLRPLTDLQVR.pdf]

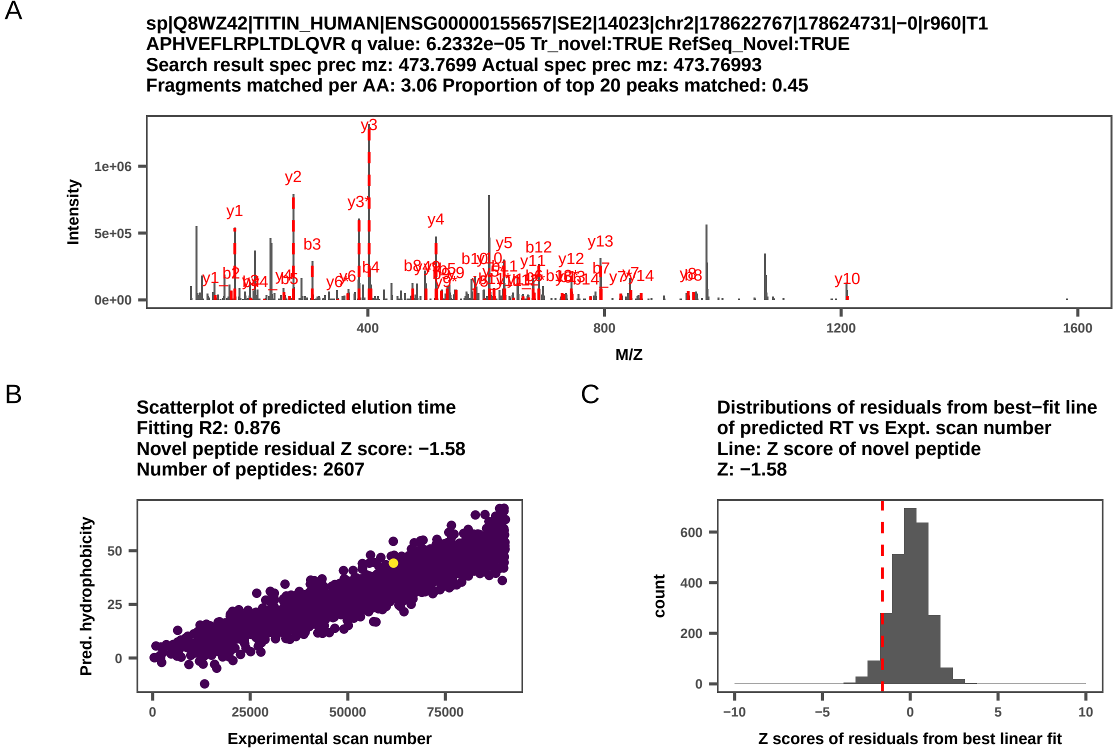
A
sp|Q8WZ42|TITIN_HUMAN|ENSG00000155657|SE2|14023|chr2|178622767|178624731|−0|r960|T1
APHVEFLRPLTDLQVR q value: 6.2332e−05 Tr_novel:TRUE RefSeq_Novel:TRUE
Search result spec prec mz: 473.7699 Actual spec prec mz: 473.76993
Fragments matched per AA: 3.06 Proportion of top 20 peaks matched: 0.45
Intensity
1e+06
5e+05
0e+00
y3
y2
y3*
y1
y4
b3
y5
b12
y13
y11
y12
b4
b7
y14
y10
400
800
1200
1600
M/Z
B
Scatterplot of predicted elution time
Fitting R2: 0.876
Novel peptide residual Z score: −1.58
Number of peptides: 2607
Pred. hydrophobicity
50
25
0
0
25000
50000
75000
Experimental scan number
C
Distributions of residuals from best−fit line
of predicted RT vs Expt. scan number
Line: Z score of novel peptide
Z: −1.58
count
600
400
200
0
−10
−5
0
5
10
Z scores of residuals from best linear fit
